# Ameloblastoma cell lines derived from different subtypes demonstrate distinct developmental patterns in a novel animal experimental model

**DOI:** 10.1590/1678-7757-2019-0558

**Published:** 2020-04-27

**Authors:** Takao FUCHIGAMI, Hajime SUZUKI, Takuya YOSHIMURA, Toshiro KIBE, Elissa CHAIRANI, Tohru KIYONO, Michiko KISHIDA, Shosei KISHIDA, Norifumi NAKAMURA

**Affiliations:** 1 Kagoshima University School of Medical and Dental Sciences Department of Oral and Maxillofacial Surgery Kagoshima Japan Kagoshima University Graduate School of Medical and Dental Sciences, Department of Oral and Maxillofacial Surgery, Kagoshima, Japan.; 2 Kagoshima University School of Medical and Dental Sciences Department of Biochemistry and Genetics Kagoshima Japan Kagoshima University Graduate School of Medical and Dental Sciences, Department of Biochemistry and Genetics, Kagoshima, Japan.; 3 National Cancer Center Research Institute Tokyo Japan National Cancer Center Research Institute, Division of Carcinogenesis and Cancer Prevention, Tokyo, Japan.

**Keywords:** Ameloblastoma, Animal model, Cell lines, Histology

## Abstract

**Objective:**

Ameloblastoma is a representative odontogenic tumor comprising several characteristic invasive forms, and its pathophysiology has not been sufficiently elucidated. A stable animal experimental model using immortalized cell lines is crucial to explain the factors causing differences among the subtypes of ameloblastoma, but this model has not yet been disclosed. In this study, a novel animal experimental model has been established, using immortalized human ameloblastoma-derived cell lines.

**Methodology:**

Ameloblastoma cells suspended in Matrigel were subcutaneously transplanted into the heads of immunodeficient mice. Two immortalized human ameloblastoma cell lines were used: AM-1 cells derived from the plexiform type and AM-3 cells derived from the follicular type. The tissues were evaluated histologically 30, 60, and 90 days after transplantation.

**Results:**

Tumor masses formed in all transplanted mice. In addition, the tumors formed in each group transplanted with different ameloblastoma cells were histologically distinct: the tumors in the group transplanted with AM-1 cells were similar to the plexiform type, and those in the group transplanted with AM-3-cells were similar to the follicular type.

**Conclusions:**

A novel, stable animal experimental model of ameloblastoma was established using two cell lines derived from different subtypes of the tumor. This model can help clarify its pathophysiology and hasten the development of new ameloblastoma treatment strategies.

## Introduction

Ameloblastoma is a representative odontogenic benign tumor showing aggressive invasion into surrounding bones.^1^ Additionally, this tumor is classified into several subtypes with distinct histological invasive growth patterns. However, the molecular mechanisms governing these characteristics are unclear. Previously, gene mutations in BRAF within the MAPK pathway and SMO within the non-MAPK pathway in ameloblastoma have been identified.^2 , 3^ These findings are very important to understand ameloblastoma and for the development of new molecular targeted therapies. However, the pathophysiology of ameloblastoma has not been sufficiently elucidated. In particular, ameloblastoma demonstrates various histological forms, such as the follicular and the plexiform types, but the causal factors for these differences remain unknown. The follicular and the plexiform types show different expression patterns in various aspects, and their properties are thought to be fundamentally different from each other.^4 - 6^ In past studies, AM-1 and AM-3 cells, which are immortalized cell lines derived from human ameloblastoma, have been chosen to elucidate the molecular mechanism of ameloblastoma invasive growth.^7 , 8^ The differences in the expression of genes such as matrix metalloproteinase have also been found, relating cell invasion of AM-1 cells to that of AM-3 cells.^8^

For cancer, a stable animal experimental model is indispensable for elucidating the pathology and pursuing new treatment strategies. This also applies to ameloblastoma, but few studies report animal experimental models of ameloblastoma. Zhang, et al.^9 , 10^ (2009,2010) established an animal experimental model of ameloblastoma consisting of subcutaneous xenografts, using primary tumor cells and tissues but not immortalized cell lines. Currently, no animal models of ameloblastoma use immortalized cells. Considering the need for experimental stability and reproducibility, an animal experimental model using immortalized ameloblastoma cell lines might be useful for researchers. The expectation is that a stable animal model will be particularly helpful for clarifying the factors underlying the differences in collective cell migration in the several invasive forms of this unique tumor. In this study, a novel animal experimental model is established by transplanting immortalized human ameloblastoma cell lines derived from different histological types into immunodeficient mice.

## Methodology

### Reagents

DMEM and Ham’s F-12 media were purchased from Nissui Corp. (Tokyo, Japan). Y-27632 was purchased from AdooQ Bioscience (Irvine, CA, USA). Hydrocortisone and insulin were purchased from Wako Pure Chemical (Osaka, Japan). Recombinant human EGF was purchased from Invitrogen Corp. (Carlsbad, CA, USA). Matrigel was purchased from Corning (New York, USA). Isoflurane was purchased from Wako Pure Chemical (Osaka, Japan). Rabbit polyclonal anti-GFP antibody was purchased from GeneTEX (Irvine, CA, USA).

### Animals

All animals were maintained and treated according to protocols established by the Division of Laboratory Animal Science of the Natural Science Center for Research and Education of Kagoshima University. The 5-week-old female BULB-c/nu immunodeficient mice used in this study were obtained from CLEA Japan (Tokyo, Japan). The mice were maintained under specific pathogen-free conditions, with constant temperature (around 27°C), and free access to food and water. All animal studies were approved by the Division of Laboratory Animal Science at the Natural Science Center for Research and Education at Kagoshima University (# D19008) and are in accordance with the Japanese government’s animal protection and management laws.

### Cells and cell culture

Two different types of ameloblastoma immortalized cell lines were used: AM-1 and AM-3. The AM-1 cells were derived from the plexiform type, whereas the AM-3 cells were derived from the follicular type.^7 , 8^ Furthermore, green fluorescence protein (GFP) expressing lentiviral vectors were constructed and transduced into ameloblastoma cells (AM-1 and AM-3) to facilitate the detection of these cells, as previously described.^11^ The GFP-labeled AM-1 and AM-3 ameloblastoma cells were maintained with F-medium (DMEM:Ham’s F-12=1:3) containing 5% fetal calf serum (FCS), insulin (10 μg/mL), Y27632 (20 μM), recombinant human EGF (0.2 μg/mL), adenine HCL (0.3 mg/mL), and hydrocortisone (2 μg/mL).

### Transplantation

The AM-1 and AM-3 cells expressing GFP were subcutaneously transplanted by injection, using a 23G needle, into the heads of immunodeficient mice under isoflurane anesthesia (3%) in the clean bench. The mice were sacrificed by cervical dislocation 30, 60, and 90 days after the transplantation, then the head tissues were collected. Ameloblastoma cells were transplanted in PBS (2×10^6^ cells/100 μl) or Matrigel (2×10^6^ cells/100 μl). Matrigel was injected without cells as a negative control. Thirty-six mice were divided randomly into groups of 3 for each evaluation point in each group. Antibiotics were not administered.

### Hematoxylin and eosin (H&E) staining and immunohistochemistry (IHC)

All samples were fixed for 48 hours in 10% formalin. Sections (4 μm thick) were prepared from paraffin-embedded blocks and stained using the EnVision+ system (Dako, Glostrup, Denmark) with an anti-GFP rabbit polyclonal antibody (1:200 dilution; GTX113617, GeneTex, San Antonio, TX, USA). Antigen retrieval was performed in a citrate buffer (pH=6.0) in water bath (95°C) for 40 minutes. Negative control sections were treated in the same manner, without the primary antibody, not showing any GFP staining. The morphology was evaluated in H&E-stained sections.

### Microscopy

Microscopic images were obtained with a conventional epifluorescence microscope (BZ-X700, KEYENCE, Tokyo, Japan).

## Results

### The tumor mass formation on the transplantation site

**Figure 1:**
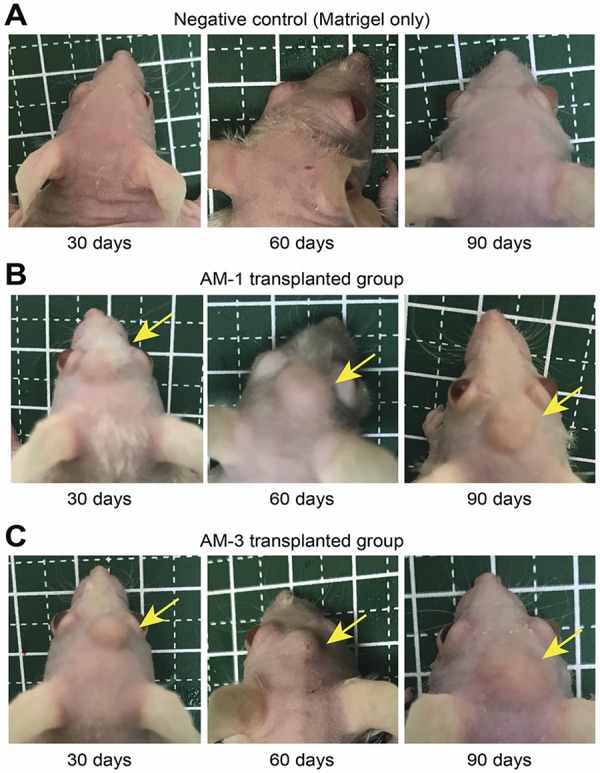
Images of the heads of mice at each time point: 30, 60, and 90 days. (A) Negative control group: transplanted with Matrigel without cells. (B) AM-1 group: transplanted with AM-1 cells with Matrigel. (C) AM-3 group: transplanted with AM-3 cells with Matrigel. Yellow arrows indicate the tumor mass

No mass formation was observed on the heads of the mice transplanted with Matrigel without cells (negative control) ( Figure 1A ), but there were tumor mass formations in the mice transplanted with AM-1 and AM-3 cells with Matrigel at all time points ( Figure 1B and C ). Moreover, cells transplanted with PBS did not survive (data not shown). The tumor size tended to increase until the mark of 60 days, after which significant changes were not observed. No mice showed any adverse events.

### Histological images of each group transplanted with different ameloblastoma cells

**Figure 2:**
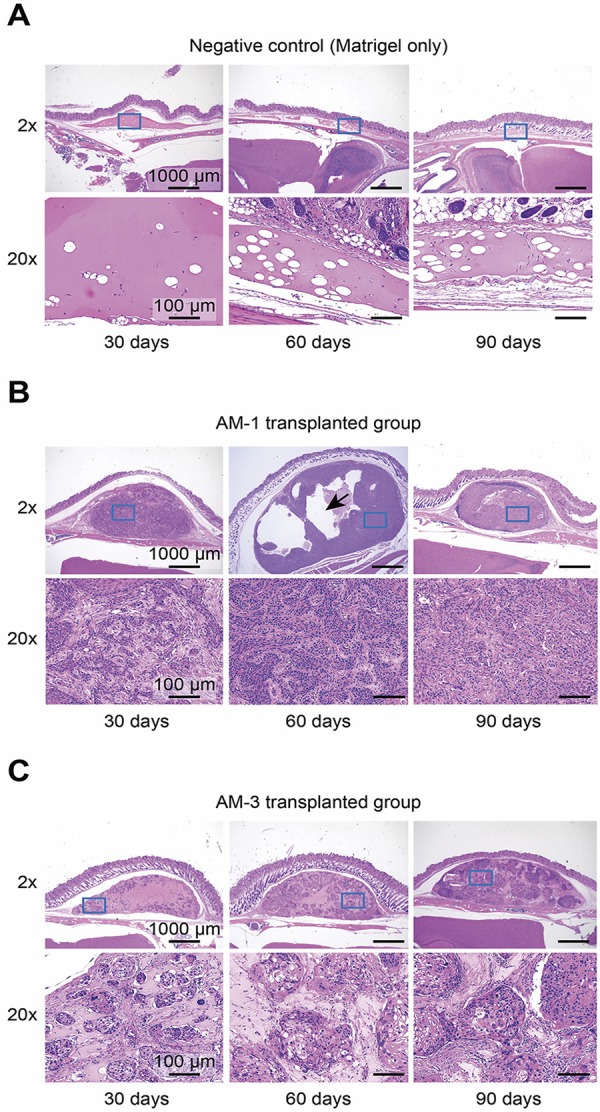
Histological images of H&E staining at each time point: 30, 60, and 90 days. (A) Negative control group: transplanted with Matrigel without cells. (B) AM-1 group: transplanted with AM-1 cells with Matrigel. (C) AM-3 group: transplanted with AM-3 cells with Matrigel. Arrow: Cyst formation at the tumor site in the AM-1 group. Lower panels show the areas marked by blue boxes in the upper panels. Magnification: Upper panels, 2×; Lower panels, 20×

Ameloblastoma cells derived from different types of the tumor were suspended in Matrigel and transplanted into mice. In the negative control group, a small mass of Matrigel without cells appeared at the transplantation site ( Figure 2A ). In contrast, the engraftment of ameloblastoma cells was found in the slides of all other mice ( Figure 2B and C ). In the AM-1 and AM-3 groups, different histological images were observed. The histologically invasive form of the AM-1-transplanted group was similar to that of the plexiform type ( Figure 2B ), and that of the AM-3-transplanted group was similar to the form of the follicular type ( Figure 2C ). Furthermore, cyst formation was observed in the transplanted lesions of the AM-1 group but not in those of the AM-3 group ( Figure 2B ).

### Evaluation of GFP expression by immunohistochemistry to confirm the presence of transplanted ameloblastoma cells

**Figure 3:**
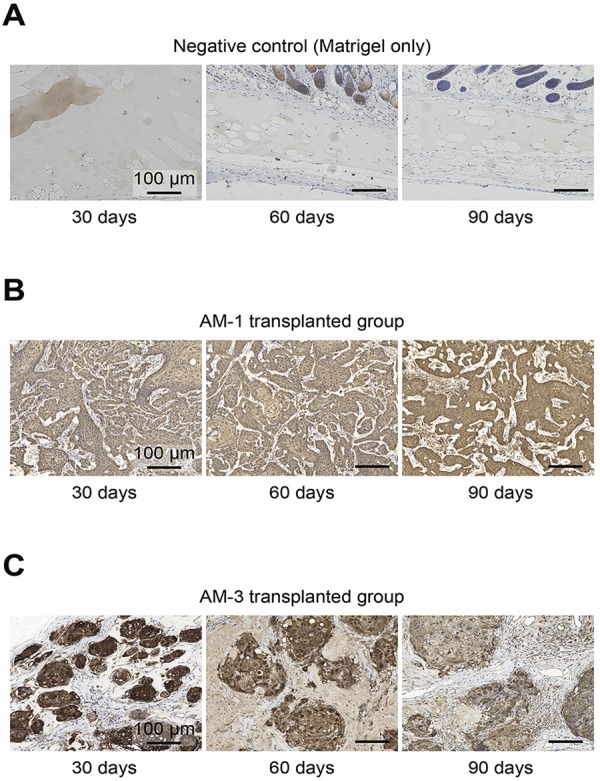
Histological images of the immunohistochemistry using the GFP antibody at each time point: 30, 60, and 90 days. (A) Negative control group: transplanted with Matrigel without cells. (B) AM-1 group: transplanted with AM-1 cells with Matrigel. (C) AM-3 group: transplanted with AM-3 cells with Matrigel. Magnification: 20×

The distribution of ameloblastoma cells was confirmed by immunohistochemistry (IHC) staining, using a GFP antibody. In the negative control group, no GFP-positive tumor cells were observed at the transplantation site ( Figure 3A ). In contrast, the tumor parenchyma was stained on all slides of ameloblastoma cell-transplanted mice ( Figure 3B and C ). Additionally, cellular components around the tumor parenchyma were not stained by the GFP antibody ( Figure 3B and C ).

## Discussion

Ameloblastoma is a tumor formed by the proliferation of odontogenic-like epithelial cells. The characteristics of this tumor are aggressive invasion into surrounding tissues and local recurrence.^1^ Therefore, wide resection of the jaw is often performed as part of its treatment.^12 , 13^ However, the pathophysiology of ameloblastoma is still reasonably unknown, and new therapeutic methods, such as molecular targeted therapy, have not been developed. The causal factors of the various histological subtypes of ameloblastoma, such as the follicular and the plexiform, are particularly difficult to investigate.

AM-1 cells derived from the plexiform type and AM-3 cells derived from the follicular type show different collective cell invasion patterns in modified three-dimensional cultures, as shown previously.^11^ Furthermore, the presence of fibroblasts affected the invasive form of ameloblastoma cells.^13^ These findings suggest the properties of ameloblastoma cells and stromal cells, including fibroblasts around tumor cells, may affect the developmental characteristics of tumor cells. Three-dimensional culture models are useful for studies targeted at specific cells, but it is difficult to mimic the actual tumor environment completely with such models. Thus, the animal experimental model is necessary to solve these problems. In this study, a novel animal experimental model of ameloblastoma was established using immortalized cell lines.

A transplant model of ameloblastoma has already been reported, Zhang, et al.^10^ (2010) transplanted human ameloblastoma cells under the kidney capsule. Moreover, transplantation models for odontogenic keratocysts are also reported.^14^ However, no reports were found of an experimental animal model using immortalized cell lines of odontogenic tumors. Numerous animal experimental models have been reported for cancer, including oral cancer, in which tumor cells were suspended in PBS or Matrigel for the transplantation. In this study, tumor cell engraftment was achieved by suspending the cells in Matrigel, a solubilized base extracted from Engelbreth-Holm-Swarm (EHS) mouse sarcoma, which includes laminin, type IV collagen, heparin sulfate proteoglycan, entactin/nidogen and several growth factors. This product is often used for cell culture and animal transplantation.^15 - 17^ Since ameloblastoma cells do not have apparent proliferative activity similar to cancer cells, the Matrigel was used as a scaffold and as a growth factor assistant besides transplantation.

In this study, an experimental model that is stable, simple, and reproducible for ameloblastoma studies was created. Furthermore, the transplantation of ameloblastoma cells at a site adjacent to the skull is beneficial for evaluating tumor-dependent effects on bone. There are reports that ameloblastoma cells promote osteoclast differentiation,^8 , 18^ and the expression of bone remodeling markers varies depending on the histological type.^19^ Hence, the effects of ameloblastoma cells on bone have to be studied in animal models.

In this study, only ameloblastoma cells were transplanted. It is not currently possible to evaluate the influence of stromal cells around tumors. Therefore, the simultaneous transplantation of ameloblastoma cells and stromal cells, such as fibroblasts and bone marrow cells, is yet to be performed in further studies. In addition, cellular components around the tumor parenchyma were not stained by IHC staining for GFP. These cellular components probably were not transformed by ameloblastoma cells but they might have migrated from the surroundings of engrafted tumors. Originally, ameloblastoma is a tumor formed in the jaw, and the environment of the bone marrow might influence its development. Even in other tumors, the bone marrow environment has been reported to play an important role in tumor development.^20^ In this study, ameloblastoma cells were subcutaneously transplanted into the heads of mice. A limitation of this study is that the experimental environment is very different from the actual developmental environment of ameloblastoma. Therefore, to investigate the behavior of ameloblastoma cells in the bone, using larger animal species, such as dogs, is necessary.

The follicular type and the plexiform type are representative histological subtypes of ameloblastoma, classified according to the structure of the tumor stroma. Some reports indicate that the follicular type has a higher recurrence rate than the plexiform type,^13^ but others indicate no clinically significant difference.^21^ In the 4^th^ edition of the WHO classification, in 2017, the difference in organization type is not regarded as important.^22^ However, the causal factors for the various histological types have not yet been elucidated, so it is suggested that these factors should be more adequately investigated. In this study, human ameloblastoma immortalized cell lines derived from two different types were successfully transplanted and engrafted into immunodeficient mice. Using this animal model of different types of ameloblastoma, previously unknown factors concerning the characteristics of ameloblastoma can be investigated, and these findings may provide important insight into the pathophysiology of ameloblastoma.

## Conclusion

In conclusion, a novel animal experimental model of ameloblastoma was established, using two cell lines derived from different subtypes of ameloblastoma. The histological images of the group transplanted with different ameloblastoma cells were similar to each origin. This animal model might help to clarify the factors affecting various forms of ameloblastoma invasion in the future.
